# Persisting Region‐Specific Microglia Activation and Synaptic Imbalance During Remission in a DSS‐Induced Colitis Model

**DOI:** 10.1002/glia.70208

**Published:** 2026-09-02

**Authors:** Lorena Morton, Luis E. Villafuerte, Alejandra P. Garza, Nina Lindemann, Elisa Wider‐Eberspächer, Dunja Bruder, Ildiko R. Dunay

**Affiliations:** ^1^ Institute of Inflammation and Neurodegeneration, Medical Faculty Otto‐von‐Guericke University Magdeburg Magdeburg Germany; ^2^ Institute of Medical Microbiology and Hospital Hygiene, Infection Immunology Group, Medical Faculty Otto‐von‐Guericke University Magdeburg Magdeburg Germany; ^3^ Helmholtz Centre for Infection Research, Immune Regulation Group Braunschweig Germany; ^4^ Center for Behavioral Brain Sciences (CBBS) Magdeburg Germany; ^5^ German Center for Mental Health (DZPG) Magdeburg Germany; ^6^ Center for Intervention and Research on Adaptive and Maladaptive Brain Circuits Underlying Mental Health (C‐I‐R‐C) Halle‐Jena‐Magdeburg Germany

**Keywords:** chronic DSS colitis, gut‐brain axis, immune memory, microglia, neuroinflammation, remission, synaptic plasticity

## Abstract

Ulcerative colitis (UC) patients experience cycles of active gut inflammation and remission, with neuropsychiatric comorbidities persisting even during clinical remission. While the dextran sulfate sodium (DSS) mouse model was previously applied to study gut‐brain interactions, those studies focused on acute protocols missing the chronic, relapsing–remitting nature of human UC, when patients continue to experience central nervous system symptoms. Thus, we employed a chronic DSS treatment regimen comprising three cycles of active intestinal inflammation followed by remission phases to investigate region‐specific microglial dynamics and their functional consequences on neuronal synapses. Transient blood–brain barrier alteration was detected during active chronic inflammation that was resolved during remission. Cortical microglia exhibited sustained iNOS‐enriched activation state during remission, which coincided with synaptic imbalance: VGLUT1+ glutamatergic synaptosomes increased significantly in remission, while VGAT+ GABAergic vesicles declined, alongside suppressed neuronal c‐fos expression. Hippocampal microglia adopted an ARG1‐dominant phenotype with enriched TREM2, P2Y12R, and F4/80 expression during remission, indicating a phagocytic, reparative state. Hippocampal synaptosome analysis revealed selective excitatory enhancement with preserved inhibitory markers. Morphological analysis confirmed region‐specific remodeling: cortical microglia displayed delayed process elaboration during remission, while hippocampal responses varied from transient (CA1) to persistent (CA3) somatic hypertrophy. These findings establish that remission from peripheral inflammation does not fully restore brain immune homeostasis. Persistent, region‐specific microglial reactivity with cortical pro‐inflammatory states associated with synaptic dysfunction and hippocampal adaptive responses preserving circuit integrity provides a mechanistic understanding for neuropsychiatric comorbidities in UC and suggests that brain‐targeted therapies may be required to address the full disease burden.

## Graphical abstract

Relapsing colitis region‐specific microglial states beyond remission. Cortical microglia remain pro‐inflammatory and synapse‐disruptive, while hippocampal microglia shift toward a reparative program that maintains neuronal circuit stability.

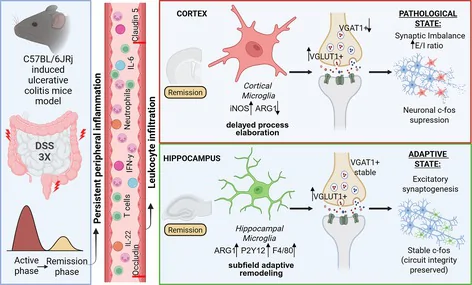

**Abbreviations**
ACC: anterior cingulate cortex
ARG1: arginase‐1
BBB: blood brain barrier
CA1: Cornu Ammonis 1
CA3: Cornu Ammonis 3
CD: cluster of differentiation
C‐Fos: Fos proto‐oncogene
CNS: central nervous system
DAI: disease activity index
DAPI: 4; 6‐diamidino‐2‐phenylindole
DG: dentate gyrus
DNA: deoxyribonucleic acid
DSS: dextran sulfate sodium
DMSO: dimethyl sulfoxide
EDTA: ethylenediaminetetraacetic acid
FACS: fluorescence‐activated cell sorting
FBS: fetal bovine serum
FCS: flow cytometry standard
FMO: fluorescent minus one
GI: gastrointestinal
HBBS: Hanks' Balanced Salt Solution
Iba1: ionized calcium binding adaptor molecule 1
IF: immunofluorescence
IL: interleukin
iNOS: inducible nitric oxide synthase
MDD: major depressive disorder
LOF: leica object file
MFI: mean fluorescence intensity
MHC‐II: Major Histocompatibility Complex‐II
NeuN: neuronal nuclei
PBS: phosphate‐buffered saline
PFC: prefrontal cortex
PFA: paraformaldehyde
PET: positron emission tomography
RNA: ribonucleic acid
RT: room temperature
TNF: tumor necrosis factor
TSPO: translocator protein
UC: ulcerative colitis
Ym1: rodent‐specific chitinase‐like protein

## 1 Background

Ulcerative colitis (UC), a chronic inflammatory bowel disease characterized by relapsing and remitting cycles of mucosal inflammation, affects millions of patients worldwide and imposes substantial burden beyond the gastrointestinal tract. Neuropsychiatric comorbidities including anxiety and depression occur in 20%–35% of UC patients (Barberio et al. 2021; Mikocka‐Walus et al. 2016; Neuendorf et al. 2016; Byrne et al. 2017), with emerging evidence of cognitive dysfunction during active inflammatory flares that persists even during clinical remission, when mucosal healing has occurred and systemic inflammation has resolved (Neuendorf et al. 2016; Bisgaard et al. 2022; Fakhfouri et al. 2024). This paradox suggests that central nervous system immune responses may outlast peripheral disease activity, yet the underlying neuroimmune mechanisms remain poorly understood. While short‐term acute DSS‐induced colitis models have established that systemic inflammation drives microglial activation, blood–brain barrier disruption, and neuroinflammation, these studies have focused almost exclusively on active disease phases (Do and Woo 2018; Han et al. 2018; Carrascal et al. 2022). Whether chronic, cyclic gut inflammation induces persistent, region‐specific microglial reprogramming that survives into remission has not been investigated.

Microglia cells exhibit remarkable functional heterogeneity across brain regions and disease contexts (Dos Santos et al. 2020; Courtney et al. 2021; Colonna and Butovsky 2017; Paolicelli et al. 2022; Tan et al. 2020; Depp et al. 2025). Traditionally classified into pro‐inflammatory (M1) and anti‐inflammatory (M2) phenotypes (Orihuela et al. 2016; Jurga et al. 2020; Guo et al. 2022; Chhor et al. 2013), microglia are now recognized to adopt diverse, context‐dependent activation states that cannot be captured by binary categorization (Paolicelli et al. 2022). Nevertheless, inducible nitric oxide synthase (iNOS) and arginase‐1 (ARG1) remain valuable functional markers: iNOS‐expressing microglia produce nitric oxide, which at high concentrations induces S‐nitrosylation of synaptic proteins and drives excitotoxicity (Nakamura and Lipton 2016; Nakamura and Lipton 2011; Nelson et al. 2003), while ARG1‐expressing microglia generate polyamines essential for dendritic spine formation and neurogenesis (Colonna and Butovsky 2017; Stratoulias et al. 2023; Matheis et al. 2020). Microglia also express a repertoire of receptors that regulate surveillance and phagocytic function, including TREM2 (lipid‐sensing and debris clearance) (Deczkowska et al. 2018; Salter and Stevens 2017; Rueda‐Carrasco et al. 2023), P2Y12R (purinergic sensing and process motility) (Salter and Stevens 2017; Li and Barres 2018; Ochocka and Kaminska 2021), and CX3CR1 (neuron–microglia communication) (Rueda‐Carrasco et al. 2023; Faust et al. 2025). Critically, microglial responses are region‐specific: cortical microglia exhibit distinct transcriptional profiles and activation kinetics compared to hippocampal microglia, reflecting differential developmental origins, local neuronal circuitry, and epigenetic landscapes (Tan et al. 2020; Grabert et al. 2016; Masuda et al. 2020; Lopes et al. 2022).

Experimental models of UC, particularly the dextran sulfate sodium (DSS)‐induced colitis model, have established that acute gut inflammation triggers systemic cytokine release, BBB modulation, and microglial activation (Do and Woo 2018; Han et al. 2018). However, the majority of studies employ single‐cycle acute DSS protocols lasting 5–7 days, which fail to recapitulate the chronic, relapsing–remitting nature of human UC. The few studies that examine repeated DSS cycles focus almost exclusively on active inflammatory phases (Valatas et al. 2013; Breynaert et al. 2013; Hoffmann et al. 2018), leaving the remission period, when patients paradoxically continue to experience neuropsychiatric symptoms, mechanistically unexplored. Moreover, prior work has assessed microglia using limited markers (typically Iba1 morphology or single cytokine measurements), precluding comprehensive phenotypic characterization. Crucially, the functional consequences of microglial activation for synaptic integrity and circuit‐level remodeling have not been investigated in the context of chronic gut inflammation. This gap is clinically relevant given that if neuropsychiatric symptoms during UC remission arise from persistent brain immune dysfunction rather than ongoing peripheral inflammation, then gut‐directed therapies, currently the standard of treatment, may be insufficient to resolve CNS comorbidities.

In this study, we employed a chronic DSS colitis protocol comprising three cycles of active intestinal inflammation followed by remission phase to model the relapsing–remitting trajectory of human UC. We hypothesized that chronic, cyclic gut inflammation would drive dissociable cortical versus hippocampal microglial adaptations. Using multimodal phenotyping, we provide a detailed characterization of regional microglial dynamics across chronic active disease and remission phases, revealing that remission does not equate to neuroimmune resolution.

## 2 Materials and Methods

### 2.1 Animal Model

**FIGURE 1:**
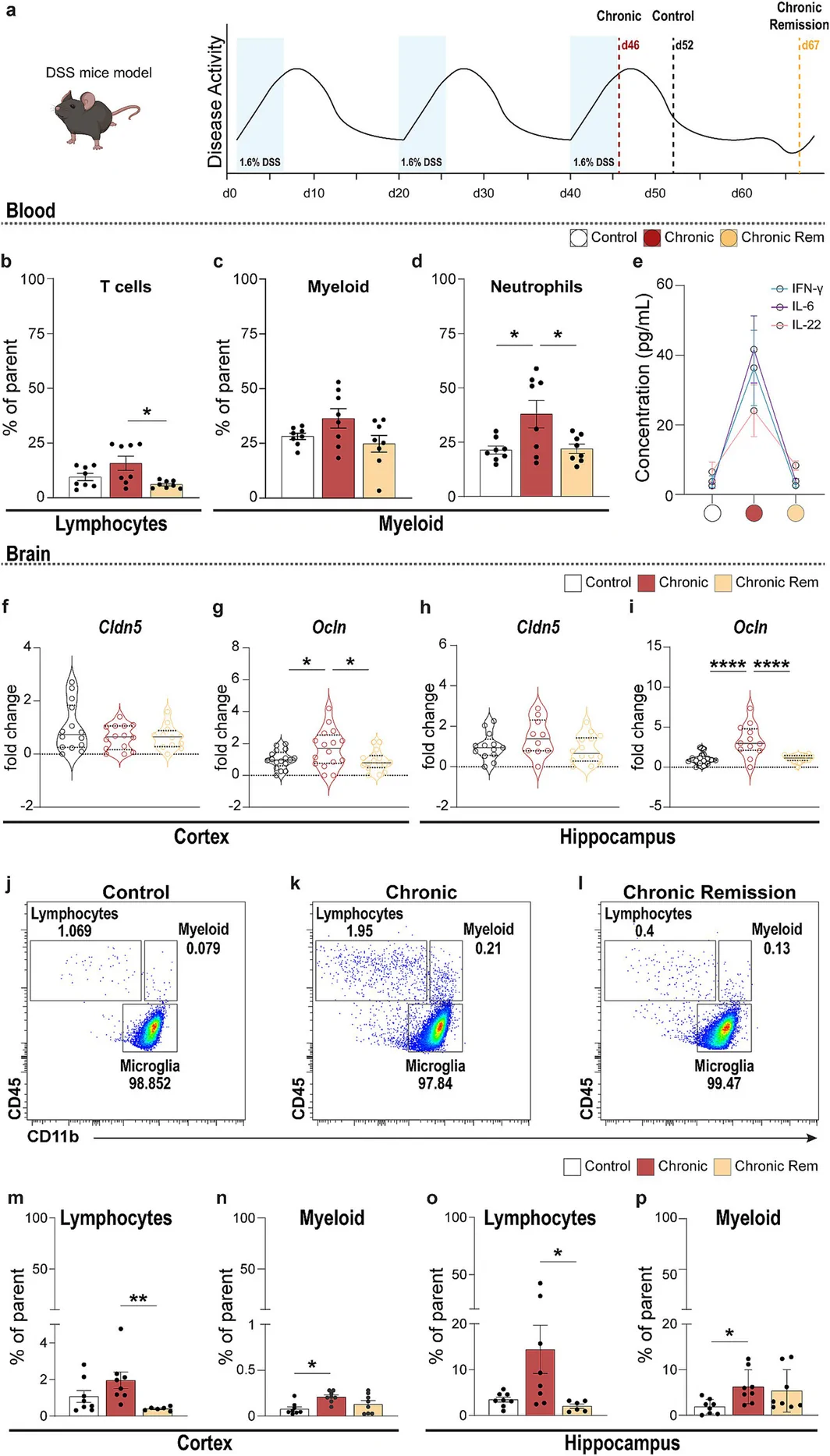
Chronic cyclic colitis induces transient blood–brain barrier modulation and region‐specific immune cell infiltration that resolve upon remission. (a) Experimental design scheme. Chronic colitis was induced in female C57BL/6JRj mice using three cycles of 1.6% dextran sulfate sodium (DSS) administration (6 days each, shaded blue) followed by 14‐day recovery periods with normal drinking water. Tissue collection occurred on Day 46 (Chronic, active inflammation) and Day 67 (Chronic Remission, 21 days post‐final DSS exposure). Control mice received normal drinking water throughout the experimental period until Day 52. (b–d) Frequency of circulating T cells (CD3^+^NK1.1^−^), myeloid (CD45^int^CD11b^+^), and neutrophils (CD11b^+^Ly6G^+^) in peripheral blood. (e) IFN‐γ, IL‐6 and IL‐22 levels in peripheral blood. (f, g) Region‐specific blood–brain barrier gene expression. Fold change in mRNA levels of tight junction proteins *Cldn5* (claudin‐5) and *Ocln* (occludin) in cortex, and (h, i) in hippocampus, normalized to *Hprt* and expressed relative to control. (j–l) Representative flow cytometry plots showing gating strategy for infiltrating CD45^hi^CD11b^−^ cells (infiltrating lymphocytes), CD45^hi^CD11b^+^ cells (infiltrating myeloid cells) and CD45^int^CD11b^+^ (resident microglia) populations. (m, n) Frequency of CD45^hi^ lymphocytes and myeloid cells in the cortex. (o, p) Frequency of CD45^hi^ lymphocytes and myeloid cells in the hippocampus. *N* = 8 mice per group; each data point represents one biological replicate; each experiment was performed twice. All data are presented as mean ± SEM. Statistical analysis was performed using one‐way ANOVA with Tukey's multiple comparisons test or Kruskal–Wallis test with Dunn's post hoc test as appropriate. *p ≤ 0.05; **p ≤ 0.01; ****p ≤ 0.0001.

All animal experiments were performed using 11 weeks‐old female C57BL/6JRj (Janvier Labs) mice. Chronic gut inflammation was induced according to a previously published DSS treatment regimen (Gelmez et al. 2022). Three different groups were studied: control group, chronic group, and chronic remission group (Figure 1a). For chronic and chronic remission groups, three consecutive cycles of DSS administration were performed. Each cycle corresponds to 1.6% weight/volume DSS oral administration for 6 days (ad libitum access), including a 14‐day interim recovery period with a supply of DSS‐free drinking water. Chronic group samples were collected on Day 46, after the last DSS administration period, and chronic remission samples were collected on Day 67 after the last recovery period.

### 2.2 Immune Cell Isolation, Staining and Measurement From Blood and Brain

Flow cytometry analysis was performed as previously described (French et al. 2022, 2019; Morton et al. 2023; Biswas et al. 2015a, 2015b). For flow cytometry, peripheral blood (400–500 μL per mouse; *n* = 8 per group) was collected in FACS Buffer. Samples were centrifuged (400 *g* for 10 min at 4°C), red blood cells were lysed using Red Blood cell Lysing Buffer (Bio Legend, 10×), and leukocytes were washed and resuspended in FACS Buffer. Cerebral cortices and hippocampi were dissected from eight brains per group and processed separately. Tissue was minced, homogenized in HBSS dissection Buffer, filtered through a 70 μm cell strainer, and centrifuged as described before our protocols.

#### 2.2.1 Surface Staining

Blood‐ and brain‐derived cells were incubated with viability dye and FC Block. Thereafter, cells were stained with specific antibodies to detect lymphoid cell markers (Table S1), myeloid cell markers (Table S2), and microglia activation markers (Table S3). After staining, cells were fixed with 4% PFA and stored overnight at 4°C in FACS Buffer. Following FACS Buffer resuspension, cells were plated and measured in the Attune NxT Flow Cytometer (French et al. 2022, 2019; Morton et al. 2023).

#### 2.2.2 Intracellular Staining

For the analysis of microglia markers, additional intracellular staining was performed in cerebral cortex and hippocampus samples. After the 4°C overnight incubation, microglia cells were resuspended in permeability buffer (Perm Wash). Cells were stained with intracellular antibodies (Arginase 1 and iNOS, Table S3) diluted in permeability buffer. Cells were washed and resuspended in FACS Buffer prior to acquisition. Following the resuspension, the plates were measured in the Attune NxT Flow Cytometer. All voltages were set based on single staining of calibration beads. FMOs controls, unstained controls and samples were recorded. The data were exported as FCS‐files and analyzed using FlowJo software (v10.5.3). Manual gating was performed to characterize myeloid cells (Figure S2a), lymphoid cells (Figure S2b) and microglia (Figure 1j–l) accordingly. Data were exported as an excel file and subsequently analyzed using GraphPad Prism 8 software (Morton et al. 2023).

### 2.3 Quantification of Serum Cytokines

#### 2.3.1 Sample Collection and Preparation

Blood was collected from mice via cardiac puncture after euthanization. Samples were allowed to clot at room temperature for 20 min and then centrifuged at 10000 rpm for 10 min at 4°C to isolate serum. Serum aliquots were stored at −80°C until analysis. Prior to assay, samples were thawed on ice and gently mixed, and samples exhibiting hemolysis were excluded.

#### 2.3.2 LEGENDplex Assay Overview

Cytokines in serum were quantified using the BioLegend LEGENDplex Mouse 12‐plex Panel (Cat. No. 741027; BioLegend, San Diego, CA, USA) according to the manufacturer's instructions, with minor modifications to reagent volumes: The assay buffer, standard, mixed beads, detection antibodies and AS‐PE were used at half concentration. This bead‐based immunoassay enables the simultaneous quantification of 12 analytes: IL‐1α, IL‐1β, IL‐6, IL‐10, IL‐12p70, IL‐17A, IL‐23, IFN‐γ, TNF‐α, MCP‐1 (CCL2), RANTES (CCL5), and MIP‐1α (CCL3). Data acquisition was performed in accordance with the manufacturer's instructions using a Sony ID7000 flow cytometer equipped with a 488 nm excitation laser and appropriate filter settings for the LEGENDplex assay. Cytokine concentrations were calculated using the LEGENDplex Data Analysis Software (BioLegend) by applying a five‐parameter logistic (5‐PL) regression model to the standard curves. Concentrations were expressed in pg/mL. Statistical analyses were performed using GraphPad Prism (v10.6.1). Data were tested for normality using the Shapiro–Wilk test. Normally distributed groups (*p* > 0.05) were compared using an unpaired *t*‐test with Welch's correction, whereas non‐normally distributed groups (*p* < 0.05) were analyzed using the Mann–Whitney *U* test. A *p* value < 0.05 was considered statistically significant.

### 2.4 Fluorescent Staining, Imaging and Analysis

Mice were perfused with PBS followed by 4% PFA. Brains were post‐fixed in 4% PFA for 24 h, cryoprotected in 30% sucrose until sinking, snap‐frozen, and stored at −80°C. Based on stereotaxic coordinates (Paxinos and Franklin 2004), cortical and hippocampal regions were sectioned using Thermo Scientific CryoStar NX50 cryostat. Free‐floating sections were incubated with primary antibodies against ARG1, iNOS, ionized calcium binding adaptor molecule 1 (Iba1), neuronal nuclei (NeuN), Fos proto‐oncogene (c‐Fos), and corresponding secondary antibodies (Table S4). All IF images were acquired using the Leica SP8 Laser Confocal Microscope. LAS X software was used to adjust specific laser power and imaging acquisition settings to obtain different sequencing images. Regions of interest included prefrontal cortex (PFC), anterior cingulate cortex (ACC), motor cortex (M1), dentate gyrus (DG), CA1, and CA3. Panoramic images were obtained at 20×, and high‐resolution images at 63× using Z‐stack acquisition. Images were exported as LOF files and processed in ImageJ with uniform contrast and brightness adjustments. Maximum intensity projections were applied to Z‐stacks. The mean fluorescence intensity (MFI) analysis, soma area, process length, and endpoints measurement from Iba1 positive cells was performed as it has been previously described (Morton et al. 2023; Young and Morrison 2018). Two brains were analyzed per experiment, and each experiment was repeated twice (8–10 fields of view per mouse). Quantitative data was used to plot the microglia soma area differences per brain region per group upon DSS‐induced colitis, as well as the MFI for Arg1 and iNOS using GraphPad Prism 8 software.

### 2.5 RT‐qPCR

Brains designated for RT‐qPCR were obtained from mice that underwent oral DSS administration cycles (Figure 1a). RT‐qPCR was performed as previously described (French et al. 2022, 2019; Morton et al. 2023, 2025). Cerebral cortex and hippocampus were processed separately. Tissue samples were homogenized in peqGOLD TriFast reagent using a benchtop homogenizer. RNA was precipitated using isopropanol, washed twice with 75% ethanol, air‐dried, and resuspended in RNase‐free water.

RNA concentration and purity were assessed by spectrophotometry using Thermo Scientific NanoDrop. Samples were analyzed using the 260/280 nm absorbance ratio of 2.0, and acceptable ratios were used for downstream analysis. The RT‐qPCR assay used for the experiments was RT‐qPCR, TaqMan Gene Assay. 15 ng RNA per sample was plated in 96‐well plates according to specific panels with genes of interest (target genes) and reference gene. Triplicate reactions were developed per sample using a real‐time PCR system for rapid cycling (LightCycler 96 Instrument). Reaction settings were selected as described before (French et al. 2022, 2019; Morton et al. 2023, 2025) Relative expression levels of target genes (*Cldn5*, *Ocln*, *Tjp1*, *Chil3*, *fos*, *Slc17a7* and *Slc32a1*; Table S5) were normalized to the reference gene *Hprt* (hypoxanthine phosphoribosyl transferase) and expressed relative to control levels. Data were analyzed using the LightCycler 96 SW Software 1.1, in which target/reference ratios were calculated. Further statistical analysis and plotting were conducted by GraphPad Prism 8 software.

### 2.6 Synaptosome Isolation, Staining and Flow Synaptometry

Cerebral cortex and hippocampus were dissected from individual mice (*n* = 8 per group; each experiment repeated twice), snap‐frozen separately in liquid nitrogen and stored at −80°C. Each animal was processed as an independent biological replicate. Synaptosomes were isolated from individual brains by differential and density gradient ultracentrifugation as previously described (Demuth et al. 2023; Steffen et al. 2025), with modifications. Briefly, each brain region was homogenized in Buffer A (10 mM HEPES pH 7.4, 0.32 M sucrose) using a glass‐Teflon Potter homogenizer. After two sequential centrifugations at 1000 × *g* (10 min, 4°C) to remove nuclei and debris, the combined supernatants from these two low‐speed spins of each individual sample were centrifuged at 12,000 × *g* (15 min, 4°C). The resulting pellet was washed once, resuspended, and loaded onto a discontinuous sucrose gradient (0.85/1.0/1.2 M). Following ultracentrifugation at 80,000 × *g* (2 h, 4°C), the synaptosomal fraction at the 1.0/1.2 M interface was collected, diluted in SET buffer (320 mM sucrose, 5 mM Tris, 1 mM EDTA), and pelleted by ultracentrifugation at 100,000 × *g* (1 h, 4°C). After a second wash (100,000 × g, 30 min, 4°C), synaptosomes were resuspended in Cryo buffer (SET +5% DMSO), aliquoted, and frozen at −80°C using a controlled‐rate freezing container.

For staining, frozen synaptosome aliquots were thawed, centrifuged, and resuspended in FoxP3 Transcription Factor Staining Buffer (eBioscience). After washing, synaptosomes were blocked and permeabilized in Permeabilization Buffer (eBioscience) containing 10% normal goat serum (NGS, ThermoFisher). Primary antibodies against synaptic proteins were incubated for 45 min at 4°C: Synaptophysin (#101308, Synaptic Systems), Synaptobrevin2 (#104318, Synaptic Systems), GluR1 (#13185, Cell Signaling Technology; #ABN421, Sigma‐Aldrich), Neuroligin2‐AF546 (#129511, Synaptic Systems), Gephyrin‐AF546 (#147011BT, Synaptic Systems), Glycine receptor‐AF546 (#146011BT, Synaptic Systems), VGLUT1 (#135011BT, Synaptic Systems), VGAT (#131011BT, Synaptic Systems). Following washing, samples were incubated with secondary antibodies: goat anti‐mouse AlexaFluor 405 (#A31553, ThermoFisher), goat anti‐rabbit AlexaFluor 488 (#ab150081, Abcam), and goat anti‐guinea pig AlexaFluor 647 (#A21450, ThermoFisher). After final washing, synaptosomes were resuspended in SET buffer containing FM^™^4‐64FX styryl dye (0.2 μg/mL, ThermoFisher) and incubated for 10 min at 4°C.

Stained synaptosomes were analyzed immediately using an Attune NxT Flow Cytometer (ThermoFisher) with 405, 488, 561, and 633 nm lasers. FM4‐64 fluorescence was used as the acquisition trigger to selectively detect membrane‐intact synaptosomal events. Samples were acquired at the lowest flow rate to minimize coincident detection. Events were gated for size (300–1000 nm) using fluorescent silica beads (Creative Diagnostics). Data were analyzed using FlowJo software (v10.10.0), with fluorescence minus one (FMO) controls used to define positive populations.

### 2.7 Statistical Analysis

Statistical analysis of microglial surface marker expression (MFI values) was performed using GraphPad Prism (v10). Individual MFI values per sample were first tested for normality using the Shapiro–Wilk test. Depending on the distribution of each dataset: Parametric data were analyzed using a one‐way ANOVA, followed by Tukey's multiple comparisons test. Nonparametric data were analyzed using a Kruskal–Wallis test, followed by Dunn's multiple comparisons test. All comparisons were conducted between the three experimental groups: Control, Chronic DSS, and Chronic Remission. Exact *p* values from each pairwise comparison (microglia heatmap) (Control vs. Chronic, Control vs. Remission, Chronic vs. Remission) are shown in Figure S3. A significance threshold of *p* < 0.05 was applied. Sample sizes were *n* = 8 per group unless otherwise stated. All data are presented as mean ± SEM, and individual sample values are plotted in bar graphs to visualize group variability and distribution.

## 3 Results

### 3.1 Chronic Colitis Induces Transient Blood–Brain Barrier Modulation and Region‐Specific Leukocyte Infiltration

To model the intermittent inflammatory flares and remission phases characteristic of UC, we implemented a chronic DSS protocol comprising three cycles of DSS exposure followed by recovery intervals (Figures 1a and S1). Multicolor flow cytometric phenotyping of peripheral blood leukocytes revealed systemic immune activation during chronic inflammation. Specifically, we observed a marked expansion of circulating lymphocytes and myeloid populations, both of which normalized during remission (Figure 1b–d), as well as increased IFN‐γ, IL‐6, and IL‐22 levels during the peak phase of chronic intestinal inflammation (Figure 1e). We found Th cells and neutrophils as the dominant systemic alterations during active disease. To assess potential CNS entry points for immune crosstalk, we analyzed the expression of tight junction‐associated genes in cortex and hippocampus. *Cldn5* remained unchanged in both regions, but *Ocln* was transiently upregulated during inflammation, with a modest increase in cortex and a pronounced elevation in hippocampus. This effect fully resolved during remission (Figure 1f–i), indicating a region‐specific and reversible barrier modulation. To determine whether chronic inflammation facilitated immune cell infiltration into the brain, we isolated CD45^+^ cells from cortex and hippocampus and gated for lymphoid and myeloid subpopulations (Figure 1j–l). Infiltrating lymphocytes and myeloid were significantly elevated in the hippocampus compared to cortex during chronic colitis, consistent with regional vulnerability. Importantly, lymphocyte frequencies declined in both regions during remission (Figure 1m,o), unlike myeloid frequencies (Figure 1n,p), suggesting that barrier modulation coincides with reduced lymphoid entry into the CNS.

### 3.2 Chronic Colitis Induces Region‐Specific Microglial Reactivity

Peripheral inflammation elicits microglial responses, yet whether chronic gut inflammation induces persistent functional reprogramming or merely transient reactivity remains unclear. We quantified microglial abundance, morphology, and surface phenotype across cortex and hippocampus throughout disease progression and remission.

**FIGURE 2:**
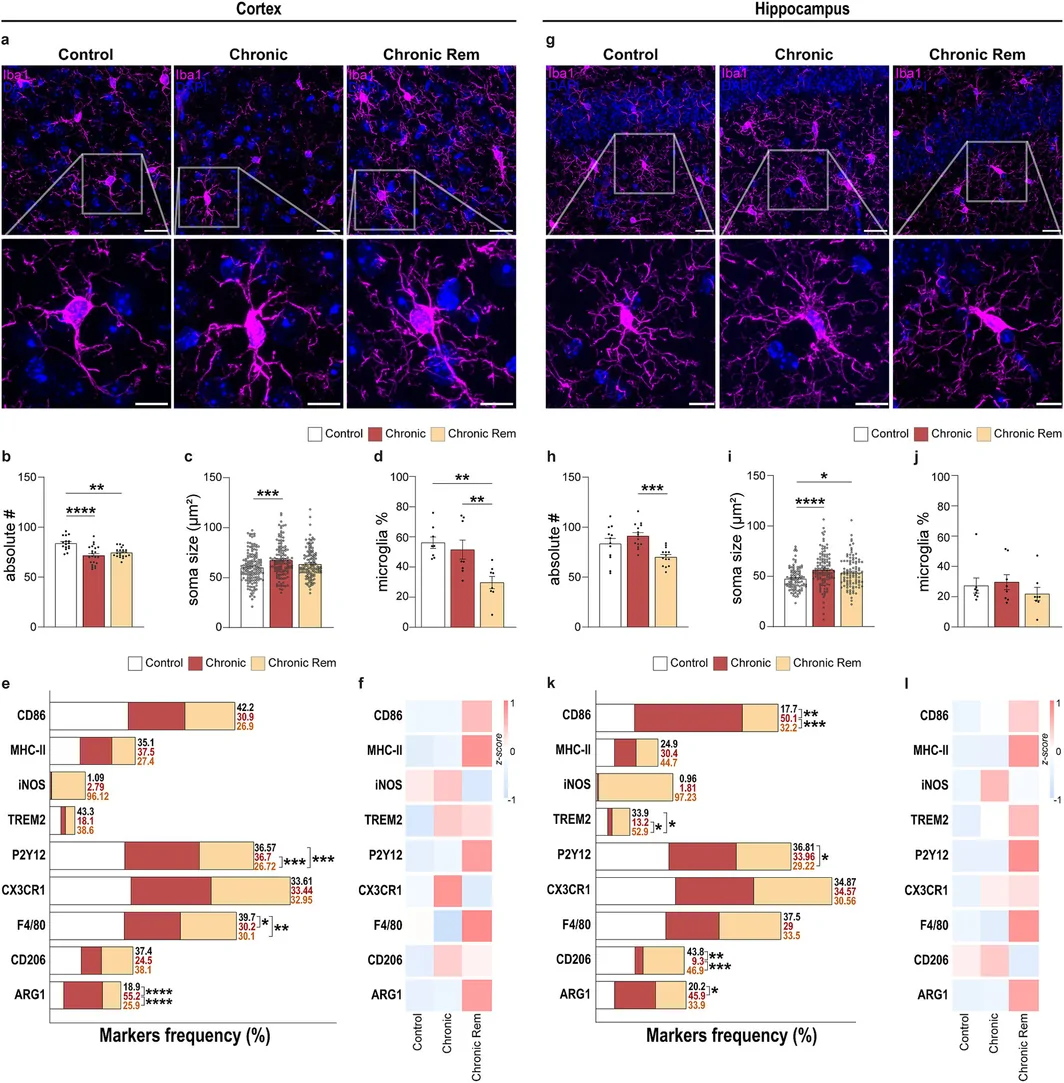
Region‐specific microglial phenotypic reprogramming persists into remission despite resolution of peripheral inflammation. (a) Representative confocal immunofluorescence images of Iba1^+^ microglia (magenta) and DAPI‐labeled nuclei (blue) in cortex and across experimental groups. Upper panels: Panoramic views showing regional microglial distribution. Lower panels: High‐magnification images (white boxes) revealing individual microglial morphology (Scale bar 63× image = 20 μm; single cell = 10 μm). (b–d) Cortical Iba1^+^ microglial quantification. (b) Absolute number of Iba1^+^ microglia per field of view and (c) Soma size (μm^2^). (d) Frequency of microglia (CD45^int^/CD11b^+^) as percentage of total CD45^+^ cells measured via flow cytometry. (e, f) Cortical microglial surface marker profiling by flow cytometry. Bar graphs showing frequency of CD45^int^/CD11b^+^ microglia expressing corresponding marker. Heatmap displaying z‐scored across groups (mean = 0, SD = 1). Red indicates above‐average expression; blue indicates below‐average expression. (g) Iba1^+^ microglia (magenta) and DAPI‐labeled nuclei (blue) in hippocampus across experimental groups. (h–j) Hippocampal microglial quantification. (h) Absolute number of Iba1^+^ microglia and (i) soma size. (j) Frequency of microglia as percentage of total CD45^+^ cells. (k, l) Hippocampal microglial surface marker profiling. (k) Bar graphs showing marker frequencies. (l) Z‐scored heatmap. See Figure S5 for raw MFI and statistics. All data are presented as mean ± SEM with individual data points. Statistical analysis: One‐way ANOVA with Tukey's post hoc (parametric) or Kruskal–Wallis with Dunn's (non‐parametric). *p ≤ 0.05; **p ≤ 0.01; ***p ≤ 0.001; ****p ≤ 0.0001.

Cortical Iba1^+^ microglia declined in absolute number during chronic colitis, a reduction that persisted through remission (Figure 2a,b). Concurrently, soma size increased significantly in both disease states (Figure 2c), while the microglial fraction within the total CD45^+^ compartment decreased (Figure 2d). Multicolor flow cytometric profiling revealed coordinated but regionally divergent shifts in activation markers (Figures 2e,f and S3). iNOS^+^ microglial frequency remained low during chronic colitis but surged dramatically in remission, while per‐cell expression intensity declined, indicating widespread low‐level iNOS induction across the population. CX3CR1^+^ microglial frequency remained stable across disease states, while expression intensity increased transiently during chronic colitis and normalized in remission, indicating reversible amplification of neuron–microglia signaling capacity. P2Y12R^+^ microglial frequency declined during chronic colitis and remained low in remission. However, per‐cell expression intensity increased selectively in remission, indicating enrichment of P2Y12R high expressing subsets. (Figures 2e,f and S3). TREM2 frequency remained stable, but expression intensity increased during chronic colitis and persisted during remission. F4/80 frequency dropped during inflammation but rebounded beyond baseline in remission, with expression intensity following a similar biphasic trajectory. ARG1^+^ microglial frequency peaked during chronic colitis and declined toward baseline in remission; however, ARG1 expression intensity per cell increased selectively in remission, establishing sustained high‐level expression in refined subsets. CD86, CD206, and MHC‐II remained largely unaltered across conditions, indicating limited modulation of classical activation markers.

Hippocampal microglia responded distinctly: absolute cell numbers remained stable during chronic colitis but declined in remission (Figure 2g,h), while soma size increased significantly during the chronic phase and remained elevated (Figure 2i,j). Activation markers (CD86, MHC‐II) showed sustained per‐cell expression despite population contraction, (Figures 2k,l and S3). Phagocytic markers exhibited remission‐phase enrichment: TREM2 frequency and intensity rebounded significantly above baseline after declining during chronic inflammation, P2Y12R intensity increased despite declining frequency, and F4/80 rose. ARG1^+^ frequency transiently expanded during chronic colitis but normalized by remission, while per‐cell intensity increased significantly in remission, indicating a shift from population expansion to sustained reparative programming. Homeostatic markers (CX3CR1, CD206) showed transient modulation with recovery by remission.

CD86^+^ microglial frequency expanded during chronic colitis and partially retracted in remission, while expression intensity increased progressively, indicating sustained per‐cell costimulatory capacity despite population contraction (Figures 2k,l and S3). MHC‐II^+^ frequency remained stable and increased intensity during remission. iNOS^+^ microglial frequency remained minimal during chronic colitis but increased in remission, while per‐cell expression intensity increased in chronic and declined in remission, mirroring the cortical pattern of widespread low‐level iNOS induction. TREM2^+^ microglial frequency declined during chronic colitis but rebounded significantly above baseline in remission, with expression intensity following the same biphasic trajectory, indicating delayed but robust upregulation of lipid‐sensing and phagocytic capacity. P2Y12R frequency declined during chronic colitis and decreased further in remission, while expression intensity remained unchanged during chronic colitis but increased significantly in remission, reflecting selective enrichment of highly expressing microglial subsets despite progressive population loss. CX3CR1^+^ microglia maintained stable frequency across states, while expression intensity rose during chronic colitis and normalized in remission, suggesting transient upregulation of neuron–microglia signaling per cell without expansion of the expressing population. F4/80 frequency remained stable during chronic colitis but rose in remission, with expression intensity increasing in remission. CD206 frequency declined during chronic colitis but recovered to baseline in remission, with expression intensity remaining stable, indicating reversible suppression of this alternative activation marker. ARG1^+^ microglial frequency increased during the chronic phase but normalized in remission, while expression intensity remained stable during chronic and increased significantly in remission, indicating transition from transient population expansion to sustained high‐level reparative programming in baseline‐sized populations.

Together, these data show that chronic colitis drives region‐specific microglial reprogramming defined by divergent kinetics of receptor expression and upregulation of the phagocytic machinery. Cortical and hippocampal microglia exhibit distinct trajectories of population dynamics and per‐cell functional amplification, reflecting differential regional vulnerability to sustained systemic inflammation.

### 3.3 Region‐Specific Microglial Morphology Reveals Altered Subfield‐Dependent Surveillance

Microglial surface marker profiling revealed region‐specific reprogramming, but whether these changes translate to altered tissue surveillance capacity and whether responses are uniform within cortical and hippocampal regions remained unclear.

**FIGURE 3:**
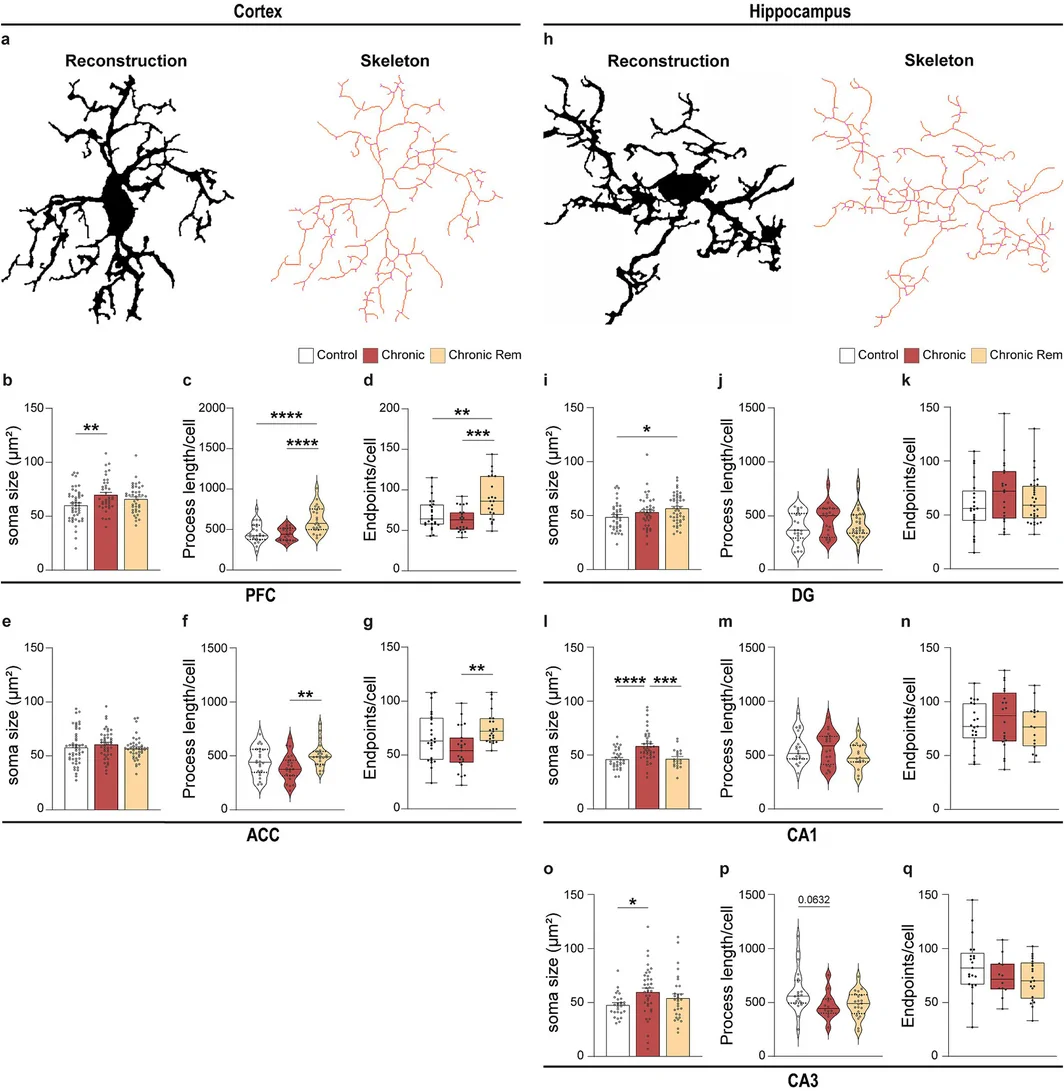
Cortical and hippocampal microglia exhibit distinct morphological remodeling trajectories with subfield‐specific adaptations. Representative binary reconstructions and skeletonized tracings of individual Iba1^+^ microglia from (a) cortex and (h) hippocampus, illustrating morphometric analysis pipeline. Reconstructions were generated from confocal Z‐stack images using ImageJ, with soma (black) and processes (branches) digitally isolated for quantification. Quantification of soma size, process length/cell and endpoints/cell from Iba1^+^ microglia of (PFC; b, c, d), anterior cingulate cortex (ACC; e, f, g), dentate gyrus (DG; i, j, k), CA1 (l, m, n), and CA3 (o, p, q) across experimental groups. Data are presented as bar graphs or violin plots (mean ± SEM) with individual data points. Statistical analysis was performed using one‐way ANOVA with Tukey's multiple comparisons test or Kruskal–Wallis test with Dunn's post hoc test as appropriate. *p ≤ 0.05; **p ≤ 0.01; ***p ≤ 0.001; ****p ≤ 0.0001.

We focused on PFC and ACC, regions critically involved in executive function and emotional regulation that are frequently impaired in UC patients (Vogt 2013; Wang et al. 2022), and hippocampal subfields CA1, CA3, and DG, which differ in neurogenic capacity, circuit connectivity, and vulnerability to systemic inflammation. High‐resolution confocal imaging and morphometric analysis of individual microglia revealed subregional heterogeneity in morphological remodeling (Figure 3). Whereas the preceding analyses characterized whole‐region, population‐level phenotypes (Figure 2), here we applied single‐cell morphometric analysis to resolve distinct subregions: PFC versus ACC, and DG versus CA1 versus CA3, a spatial resolution not attainable by flow cytometry.

Cortical microglia displayed region‐specific morphological remodeling characterized by delayed process elaboration. In PFC, soma size increased significantly during chronic colitis (Figure 3b), while process length showed a non‐significant decline during chronic colitis followed by significant elongation in remission (Figure 3c), and endpoints per cell increased selectively in remission (Figure 3d). This pattern, acute somatic hypertrophy followed by delayed process extension and branching, indicates initial activation transitioning to enhanced surveillance capacity. ACC microglia exhibited stable soma size across disease states (Figure 3e), with process length remaining unchanged during chronic colitis but increasing significantly in remission (Figure 3f), alongside elevated endpoint density (Figure 3g), suggesting region‐specific recruitment of arbor elaboration during the post‐inflammatory phase.

Hippocampal microglia displayed subfield‐specific morphological responses with distinct temporal dynamics. DG microglia showed stable soma size during chronic colitis, with significant enlargement emerging only in remission relative to control (Figure 3i), while process length and branching complexity remained unchanged throughout (Figure 3j,k). CA1 microglia showed transient soma enlargement that resolved in remission (Figure 3l), without changes in process length or endpoint density (Figure 3m,n), suggesting a transient and reversible activation state. In contrast, CA3 microglia exhibited persistent soma enlargement (Figure 3o), a trend toward shorter processes (Figure 3p), and stable endpoints (Figure 3q), indicating persistent morphological activation.

This morphological profile shows that chronic colitis drives temporally and spatially distinct microglial remodeling where cortical microglia undergo delayed process elaboration during remission, while hippocampal responses vary from transient (CA1) to persistent (CA3) somatic hypertrophy. These divergent patterns indicate subregion‐specific adaptations in microglial surveillance architecture.

### 3.4 Cortical Microglia Sustain an iNOS‐Enriched State Coinciding With Excitatory‐Inhibitory Synaptic Imbalance

Microglia shape neural circuit integrity by adapting their effector profile to environmental cues. Having identified regionally divergent microglial reprogramming during chronic colitis, we next investigated two key axes of effector output: (i) inflammatory polarization and (ii) microglial‐synapse interactions. We used iNOS and ARG1 as canonical markers of classically and alternatively activated phenotypes, respectively, and extended our analysis to synaptosome profiling and regional gene expression to dissect downstream consequences of microglial remodeling across brain compartments.

**FIGURE 4:**
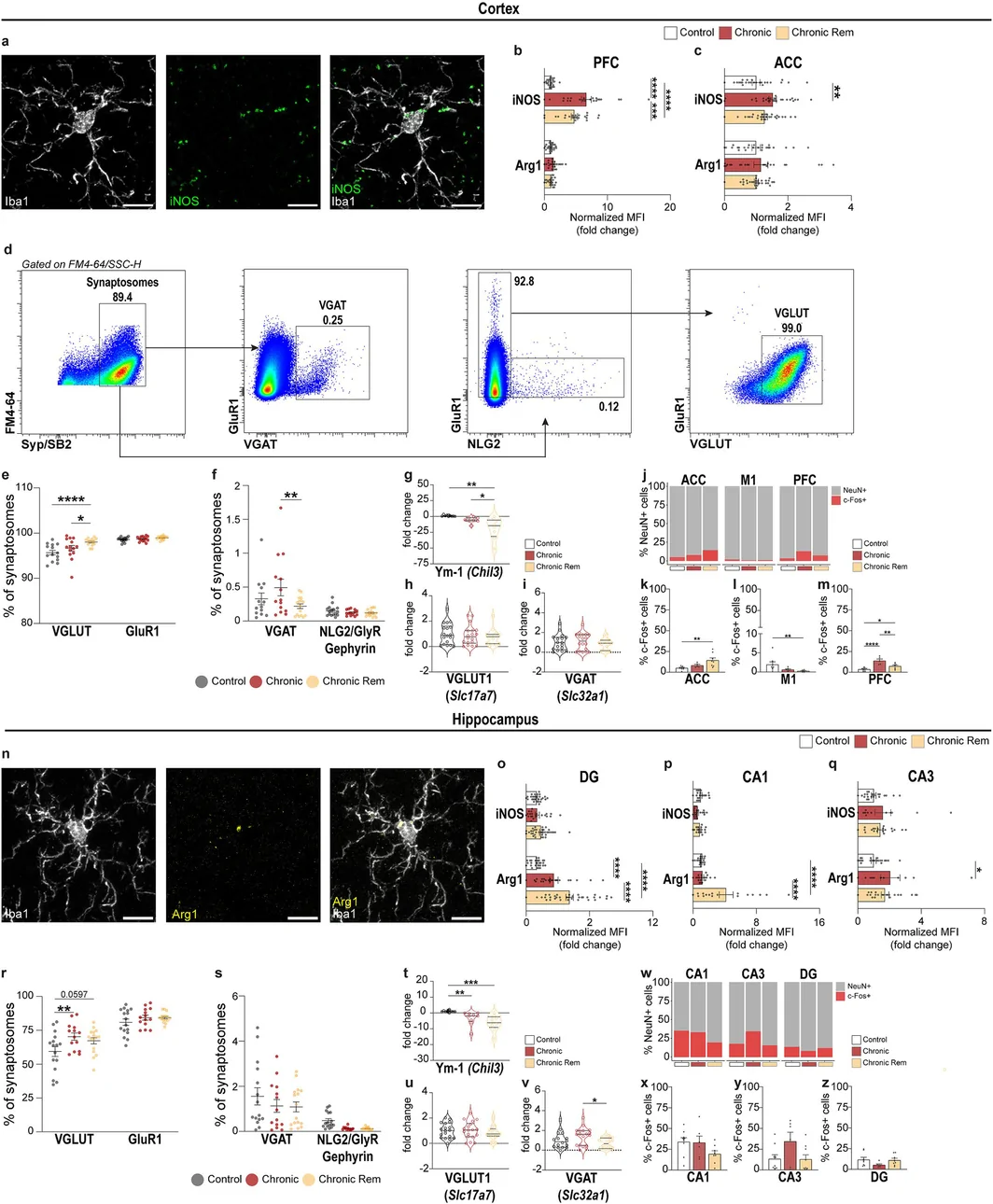
Divergent microglial polarization associates with region‐specific synaptic remodeling, cortical excitatory‐inhibitory imbalance and hippocampal adaptive plasticity. (a) Representative confocal IF images of cortical microglia showing Iba1 (gray), iNOS (green), and merged channels (right). Individual microglial cell showing co‐localization of Iba1 and iNOS. Scale bar = 10 μm. (b, c) Cortical microglial polarization. Mean fluorescence intensity (MFI) of iNOS and ARG1 in Iba1^+^ microglia from (b) prefrontal cortex (PFC) and (c) anterior cingulate cortex (ACC). (d) Synaptosomes were first identified by size (300–1000 nm, Suppl. Fig. 4f) and FM4‐64FX^+^ membrane staining (left panel), then selected for co‐expression of the pan‐synaptic vesicle markers synaptophysin and synaptobrevin‐2 (Syp/SB2^+^). From the Syp/SB2^+^ population, excitatory and inhibitory postsynaptic densities were discriminated using the postsynaptic receptor subunit GluR1 (AMPA receptor subunit 1) versus the inhibitory postsynaptic scaffold proteins NLG2 (neuroligin‐2), gephyrin, and glycine receptor, Suppl. Fig.4f; within the GluR1^+^ population, the presynaptic vesicular glutamate transporter VGLUT1 was quantified. In parallel, the presynaptic vesicular GABA transporter VGAT was quantified directly from the Syp/SB2^+^ population. (e) Frequency of VGLUT1^+^ glutamatergic synaptosomes and GluR1^+^ postsynaptic densities. (f) Frequency of VGAT^+^ GABAergic presynaptic vesicles and inhibitory postsynaptic scaffolds (NLG2, gephyrin, glycine receptor). Cortical gene expression of (g) *Chil3* (Ym‐1, alternative activation marker), (h) *Slc17a7* (VGLUT1), and (i) *Slc32a1* (VGAT) fold change in mRNA levels normalized to *Hprt*. (j–m) Frequency of cortical c‐Fos immunofluorescence co‐labeled with NeuN from (k) ACC, (l) motor cortex 1 (M1), and (m) PFC. (n) Representative hippocampal microglial showing Iba1 (gray), ARG1 (yellow) and merged channels. Scale bar = 10 μm. MFI of iNOS and ARG1 in (o) dentate gyrus (DG), (p) CA1, and (q) CA3. (r, s) Hippocampal synaptosome profiling. (t–v) Hippocampal gene expression. (w–z) Hippocampal c‐Fos immunofluorescence co‐labeled with NeuN. Individual values shown in the graphs represent mean ± SEM. Statistical analysis was performed using one‐way ANOVA with Tukey's multiple comparisons test or Kruskal–Wallis test with Dunn's post hoc test as appropriate. *p ≤ 0.05; **p ≤ 0.01; ***p ≤ 0.001; ****p ≤ 0.0001.

Immunofluorescent validation revealed region‐specific microglial polarization patterns. In PFC, iNOS expression increased significantly during chronic colitis and remained elevated in remission, though reduced from peak levels, while ARG1 remained unchanged (Figure 4a,b). ACC microglia exhibited sustained iNOS upregulation in both chronic and remission phases without ARG1 induction (Figure 4c). These findings confirm that cortical microglia maintain iNOS‐enriched signatures even during peripheral disease remission.

To determine whether sustained cortical microglial pro‐inflammatory polarization impacts synaptic integrity, we isolated synaptosomes, which are purified synaptic terminals retaining presynaptic vesicles and postsynaptic densities (Whittaker et al. 1964), from cortex and hippocampus and performed multicolor flow cytometric analysis (Gajera et al. 2019; Tieze et al. 2022; Postupna et al. 2014) of glutamatergic and GABAergic markers (Düsedau et al. 2025) (Figure 4d). Cortical synaptosome analysis revealed progressive excitatory‐inhibitory divergence across disease phases. VGLUT1^+^ glutamatergic synaptosomes, representing presynaptic vesicular glutamate transporters, increased significantly in remission compared to both control and chronic conditions (Figure 4e), indicating amplified glutamatergic transmission capacity. This increase occurred without corresponding changes in postsynaptic GluR1 (AMPA receptor subunit 1) density, suggesting presynaptic remodeling without homeostatic postsynaptic scaling.

Conversely, VGAT^+^ GABAergic presynaptic vesicles showed nonsignificant elevation during chronic colitis but declined significantly from chronic colitis to remission (Figure 4f), establishing a temporal pattern wherein inhibitory synaptic components fail to maintain compensatory upregulation as disease transitions to remission. Critically, postsynaptic inhibitory scaffolds including NLG2 (neuroligin‐2), gephyrin, and glycine receptors remained unchanged across all conditions (Figures 4f and S4d), indicating that inhibitory synapse loss is driven by presynaptic terminal retraction rather than postsynaptic disassembly.

To determine whether synaptic protein changes reflected transcriptional remodeling or posttranslational regulation, we quantified gene expression of microglial phenotype markers, neuronal activity markers, and synaptic transporter genes in cortex (Figure 4g–i). Transcriptional profiling revealed dissociation between protein and mRNA expression patterns. Chil3 (Ym‐1/CHI3L3), a canonical alternative activation marker, decreased significantly in remission below both chronic and control levels (Figure 4g), indicating that despite synaptosome‐level evidence of ongoing remodeling, microglia do not adopt a classical M2 reparative transcriptional program.

Neuronal activity assessed via bulk c‐fos qPCR (Figure S4b) showed an overall decline in cortical tissue during chronic colitis and remission. To resolve the subregional basis of this signal, we performed c‐fos immunofluorescence co‐labeled with NeuN across PFC, ACC, and M1 (Figures 4j–m and S5a). This revealed c‐Fos^+^ neuron density increased significantly in PFC and ACC, indicating selective neuronal hyperactivation in executive and affective cortical regions. In contrast, M1 showed a significant reduction in c‐Fos^+^ neurons during both phases, accounting for the bulk qPCR suppression. The elevation of c‐Fos^+^ neurons in PFC and ACC in the context of VGLUT1↑ and VGAT↓ is mechanistically coherent with amplified excitatory drive and diminished inhibitory tone, consistent with an excitotoxic overactivation state.

Synaptic transporter gene expression (*Slc17a7*/VGLUT1, *Slc32a1*/VGAT) remained stable across conditions (Figure 4h,i), indicating that synaptosome‐level protein changes arise from posttranscriptional mechanisms, including altered protein trafficking, vesicle recycling, or synapse‐specific protein stabilization, rather than bulk transcriptional regulation. This mRNA‐protein dissociation suggests localized, synapse‐autonomous control of neurotransmitter release machinery.

### 3.5 Hippocampal Microglia Adopt ARG1‐Dominant Phenotypes Associated With Excitatory Synaptogenesis and Preserved Inhibitory Tone

Hippocampal microglia displayed distinct polarization patterns characterized by regional ARG1 dominance without iNOS induction (Figure 4n–q). DG microglia showed no iNOS changes but robust ARG1 upregulation that intensified progressively from chronic colitis to remission (Figure 4o). CA1 microglia similarly lacked iNOS induction, while ARG1 expression rose in remission, surpassing both chronic colitis and control levels (Figure 4p). CA3 microglia exhibited sustained ARG1 elevation without iNOS changes, with expression plateauing from chronic colitis through remission (Figure 4q).

Hippocampal synaptosome analysis revealed a pattern characterized by excitatory enhancement without inhibitory compromise (Figure 4r,s). VGLUT1^+^ glutamatergic synaptosomes increased significantly during chronic colitis and remained elevated through remission (Figure 4r), showing sustained potentiation of excitatory synaptic transmission capacity. As in cortex, postsynaptic GluR1 density remained unchanged, suggesting that excitatory synaptogenesis occurs via presynaptic vesicle accumulation or terminal elaboration without corresponding postsynaptic receptor insertion, a pattern consistent with increased release probability or presynaptic structural plasticity.

In contrast to cortex, GABAergic markers remained stable at the protein level across all disease phases: VGAT^+^ presynaptic vesicles and postsynaptic inhibitory scaffolds, NLG2, gephyrin, and glycine receptors showed no significant changes (Figure 4s). This preservation of inhibitory synaptic machinery, occurring alongside excitatory enhancement, suggests that hippocampal circuits undergo adaptive synaptic remodeling rather than pathological imbalance.

Transcriptional profiling in hippocampus revealed temporal dissociation between mRNA and protein expression, indicating multi‐level regulatory control. *Chil3* decreased progressively from chronic to remission (Figure 4t), mirroring cortical patterns and confirming that sustained synaptic remodeling occurs independently of classical M2 microglial transcriptional activation. Bulk neuronal activity assessed via c‐fos expression remained stable across all conditions (Figure S4d). Immunofluorescent quantification of c‐Fos^+^ neurons across hippocampal subfields (CA1, CA3, DG) (Figure 4w–z and S5b) confirmed preserved neuronal activity across all groups, consistent with maintained circuit integrity in the context of adaptive microglial remodeling.

Glutamatergic transporter gene expression (*Slc17a7*/VGLUT1) remained unchanged (Figure 4u), indicating that elevated VGLUT1 protein at synaptosomes reflects posttranscriptional upregulation, potentially via enhanced mRNA translation, protein stabilization, or preferential trafficking to synaptic terminals. Notably, inhibitory transporter gene expression (Slc32a1/VGAT) declined significantly in remission (Figure 4v), despite stable VGAT protein levels at synaptosomes.

## 4 Discussion

We demonstrate that both the active phase of chronic colitis and the remission state drive region‐specific and phase‐dependent microglial phenotype characterized by dissociable cortical versus hippocampal trajectories. Cortical microglia in prefrontal and anterior cingulate regions sustain iNOS‐enriched expression into remission, which coincides with excitatory‐inhibitory synaptic imbalance, glutamatergic transporter upregulation, and suppressed neuronal activity. In contrast, hippocampal microglia across the DG, CA1, and CA3 subfields adopt a phagocytic, ARG1‐dominant phenotype enriched in TREM2 and P2Y12R, associated with enhanced excitatory synaptogenesis but preserved inhibitory tone. Critically, these regional adaptations persist after peripheral inflammation has resolved, indicating that remission from gut disease does not restore immune homeostasis in the central nervous system. These findings establish that neuroinflammation in UC is not merely reactive to peripheral immune signals but involves intrinsic, region‐specific microglial memory states that outlast triggering stimuli. We propose that sustained cortical iNOS‐enriched microglia contribute to the executive dysfunction, anhedonia, and anxiety prevalent in UC patients during remission, while adaptive hippocampal responses preserve spatial cognitive function despite affective disruption.

We emphasize that iNOS and ARG1 are employed here as functional effector markers reflecting specific biochemical outputs, nitric oxide production and polyamine synthesis, respectively. These enzymes were selected not to classify microglia into binary categories but because their products have direct mechanistically defined consequences for synaptic integrity: nitric oxide at sustained concentrations induces S‐nitrosylation of synaptic proteins and promotes inhibitory terminal vulnerability (Nakamura and Lipton 2016; Nakamura and Lipton 2011; Nelson et al. 2003), while arginase‐derived polyamines support dendritic spine formation and synaptic maturation (Colonna and Butovsky 2017; Stratoulias et al. 2023; Matheis et al. 2020). The co‐detection of both markers at the protein level, alongside a nine‐marker flow cytometry panel encompassing TREM2, P2Y12R, CX3CR1, F4/80, CD86, CD206, ARG1, iNOS and MHC‐II, was designed to resolve activation states beyond binary classification; the divergent kinetics of these markers across disease phases argue against a simple two‐state model and reflect the context‐dependent heterogeneity now recognized as characteristic of microglial biology (Paolicelli et al. 2022; Depp et al. 2025).

The persistence of cortical iNOS^+^ microglia into remission, despite resolution of peripheral inflammation and blood–brain barrier normalization, suggests epigenetic or cell‐autonomous reprogramming. Several mechanisms may sustain this iNOS‐enriched activation state. First, trained immunity: repeated DSS cycles may induce histone modifications (H3K4me3, H3K27ac) at inflammatory gene loci in cortical microglia, lowering activation thresholds for subsequent stimuli (Neher and Cunningham 2019; Zhang et al. 2022; Huang et al. 2023; Tiwari et al. 2024). Second, continued by the transient barrier dysfunction: while *Ocln* expression normalized and *Cldn5* and *Tjp1* (ZO‐1) remained unchanged (Figure S4), we did not perform functional permeability assays (FITC‐dextran), leaving open the possibility of residual paracellular leak permitting gut‐derived mediators (LPS, short‐chain fatty acids) to access cortical parenchyma. However, prior work from our group has demonstrated concordance between tight‐junction gene expression and FITC‐dextran–based permeability measurements in neuroinflammatory contexts (Figueiredo et al. 2022). In fact, we have shown earlier that intestinal dysbiosis is clearly initialized by active periods of DSS‐dosage in which each period has a cumulative progressive impact on microbiota composition (Gelmez et al. 2022). Intestinal dysbiosis in combination with increased epithelial leakiness in the colon may have profound effects on the composition and systemic distribution of gut microbial mediators. Gut‐derived LPS, a potent TLR4 agonist, represents a particularly well‐characterized route by which intestinal dysbiosis may signal to the brain. Circulating LPS activates TLR4 expressed on cerebral endothelial cells and brain‐resident microglia, triggering NF‐κB–dependent transcription of pro‐inflammatory cytokines including IL‐1β, IL‐6, and TNF‐α, a pathway documented in both systemic endotoxaemia and IBD associated neuroinflammation models (Fakhfouri et al. 2024; Han et al. 2018; Lehnard et al. 2002). Beyond LPS, short‐chain fatty acids generated by the dysbiotic gut microbiome can cross the BBB via monocarboxylate transporters and directly modulate microglial activation state and histone deacetylase activity (Erny et al. 2015; Braniste et al. 2014). Moreover, gut microbiota composition has been shown to regulate basal microglial transcriptional programs and morphology under homeostatic conditions (Erny et al. 2015), implying that the cumulative dysbiosis we observe across DSS cycles (Gelmez et al. 2022) may reprogram microglial responsiveness independently of acute cytokine signaling. Therefore, gut‐derived microbial mediators including but not limited to extracellular vesicle cargo, constitute a plausible and mechanistically supported route for sustained cortical microglial activation during peripheral remission.

Third, perivascular macrophage persistence: CD45^hi^ infiltrating cells declined but did not fully normalize in cortex, and these cells may maintain a pro‐inflammatory niche via IL‐1β and TNF‐α secretion. The consequence of sustained cortical iNOS expression was shown in our synaptic and transcriptional data. Nitric oxide, the product of iNOS enzymatic activity, is a potent regulator of synaptic function: at low concentrations it modulates LTP via cGMP signaling, but at high concentrations it induces S‐nitrosylation of synaptic proteins, impairing NMDA receptor function (Nakamura and Lipton 2016; Nakamura and Lipton 2011) and promoting GABAergic terminal retraction. Consistent with this, we observed increased VGLUT1^+^ glutamatergic synaptosomes in remission in cortex, with declining VGAT^+^ GABAergic vesicles, indicating selective loss of inhibitory presynaptic terminals. Post synaptic scaffolds (gephyrin, NLG2) remained intact, suggesting that inhibitory synapse loss is driven by presynaptic pruning rather than postsynaptic disassembly, a pattern consistent with complement‐mediated (Stephan et al. 2012; Lui et al. 2016; Gomez‐Arboledas et al. 2018) or nitric oxide‐mediated selective elimination. Cellular resolution of c‐Fos resolves what appeared paradoxical in bulk qPCR measurements. PFC and ACC neurons are not silenced but selectively hyperactivated, with c‐Fos^+^ neuron density significantly elevated during chronic colitis and sustained into remission. This hyperactivation in the face of increased glutamatergic drive (VGLUT1↑) and diminished inhibitory tone (VGAT↓) is mechanistically coherent and consistent with an excitotoxic overexcitation state in which chronic calcium influx may drive mitochondrial dysfunction and transcriptional dysregulation (Mira and Cerpa 2021; Dickey and La Spada 2018). This interpretation aligns with findings in chronic pain models, where sustained microglial activation produces neuronal sensitization, elevated transmitter release without corresponding increases in action potential firing (Beggs and Salter 2010; Huang et al. 2013). Clinically, PFC and ACC dysfunction manifests as executive deficits, decision‐making impairments, anhedonia, and anxiety (Pizzagalli and Roberts 2022; Drevets et al. 2008), largely resembling the neuropsychiatric profile observed in UC patients during remission. Our findings suggest that targeting cortical microglia may represent a therapeutic avenue independent of gut‐directed therapies.

Hippocampal microglia responded to chronic inflammation with a fundamentally different strategy: transient population expansion of ARG1^+^ cells during active colitis, followed by selective enrichment of ARG1^+^, TREM2^+^, P2Y12R^+^, F4/80^+^ subsets during remission. This phenotype is consistent with a phagocytic, reparative state optimized for synaptic refinement and neurogenic support (Colonna and Butovsky 2017; Matheis et al. 2020; Polis et al. 2019). ARG1 catabolizes L‐arginine to produce ornithine and polyamines, which are essential for dendritic spine formation, synaptic maturation, and neuronal survival. In vitro, ARG1^+^ microglia promote hippocampal neurogenesis via IL‐4 secretion, which activates STAT6 signaling in neural stem cells (Zhang et al. 2021; Feng et al. 2019), and enhance Aβ clearance in neuroinflammation models via phagocytic uptake. TREM2, a lipid‐sensing receptor was upregulated in our remission hippocampal microglia, mediates phagocytosis of damaged myelin and lipid‐rich debris (Wang et al. 2015; Nugent et al. 2020; Cignarella et al. 2020), preventing accumulation that would otherwise drive sustained chronic inflammation. P2Y12R, the microglial purinergic receptor that mediates process extension toward sites of ATP release (Li and Barres 2018; Koizumi et al. 2013; Ohsawa et al. 2007), was suppressed in frequency but enriched in per‐cell expression intensity, indicating that a smaller population of highly motile microglia maintains surveillance capacity. The output of this phenotype is evident in our synaptic data: VGLUT1^+^ glutamatergic synaptosomes increased significantly during chronic colitis and remained elevated in remission, while GABAergic markers remained stable at the protein level. Critically, VGAT mRNA declined in the hippocampus during remission, but VGAT protein did not, suggesting either translational buffering, prolonged protein half‐life, or cell‐type‐specific expression shifts (e.g., interneuron subpopulations differentially affected). We interpret the sustained VGLUT elevation not as pathological hyperexcitability but as adaptive synaptic remodeling: ARG1^+^ microglia may selectively prune weak or damaged excitatory synapses while supporting maturation of functionally competent synapses, resulting in net synaptogenesis with refined connectivity. The absence of iNOS induction in hippocampus, despite comparable peripheral inflammation exposure, suggests regional differences in microglial transcriptional programs, possibly related to local IL‐4/IL‐13 signaling from infiltrating immune cells, which we detected in hippocampus more than in cortex, or to intrinsic epigenetic differences between cortical and hippocampal microglia. Clinically, preserved hippocampal function may explain why UC patients retain spatial memory and declarative learning capacity despite profound affective disruption. However, sustained microglial phagocytic activity may have long‐term costs: excessive synaptic pruning has been implicated in cognitive decline in aging and neurodegenerative disease (Lui et al. 2016; Schafer and Stevens 2013; Hong et al. 2016; Paolicelli et al. 2011). Whether ARG1^+^ hippocampal microglia transitions from adaptive to maladaptive in prolonged UC remains to be determined.

The persistence of region‐specific microglial reprogramming into remission, when peripheral inflammation, systemic cytokines, and barrier dysfunction have resolved, challenges the prevailing model that neuroinflammation in UC is passively driven by peripheral immune signals (Fung et al. 2017; Agirman et al. 2021; Zonis et al. 2015). Instead, our findings support a model of microglial immune memory, wherein repeated inflammatory exposures induce stable epigenetic, transcriptional, or metabolic reprogramming that persists autonomously. This parallels trained immunity in peripheral macrophages, where β‐glucan or BCG exposure induces H3K4me3 marks at inflammatory gene promoters, rendering cells hyperresponsive to subsequent stimuli for months (Netea et al. 2016; Arts et al. 2016; Kleinnijenhuis et al. 2012). In microglia, trained immunity has been demonstrated in models of chronic stress and neuroinflammation, where initial insults prime exaggerated responses to secondary challenges (Wendeln et al. 2018; Zhang et al. 2025). Critically, microglial trained immunity can be both protective (enhanced pathogen clearance) and detrimental (exaggerated neuroinflammation). In our model, cortical microglia appear maladaptively trained, sustaining iNOS expression associated with synaptic imbalance, while hippocampal microglia exhibit adaptive training, maintaining phagocytic capacity that supports circuit refinement. The clinical implications are if neuropsychiatric symptoms in UC remission are driven by intrinsic brain immune memory rather than ongoing peripheral inflammation, then gut‐directed therapies (anti‐TNF biologics, immunosuppressants) may be insufficient to resolve CNS comorbidities. Instead, brain‐penetrant anti‐inflammatory strategies targeting microglia directly, such as CSF1R inhibitors (to deplete and repopulate microglia) (Spangenberg et al. 2019; Henry et al. 2020; Green et al. 2020; Nissen et al. 2018), TREM2 agonists (to shift toward phagocytic states) (Morenas‐Rodríguez et al. 2019; Xue et al. 2022), or iNOS inhibitors (to suppress nitrosative stress) (Pannu and Singh 2006; Broom et al. 2011), may be required. Alternatively, interventions during active flares that prevent microglial memory formation (e.g., minocycline Wang et al. 2016; Garrido‐Mesa et al. 2013; Cai et al. 2013), IL‐4 modulation (Zhang et al. 2021; Feng et al. 2019) may have prophylactic benefit for subsequent remission phases. Our findings also suggest that remission should not be defined solely by mucosal healing and symptom resolution but should incorporate neuroimmune biomarkers (e.g., serum neurofilament light chain [Gaetani et al. 2019; Gisslén et al. 2016; Kuhle et al. 2016]), CSF cytokines, and PET imaging of microglial activation using TSPO ligands (Dupont et al. 2017; Airas et al. 2015) to capture central nervous system disease activity.

Collectively, these data establish that chronic colitis drives region‐specific synaptic remodeling with divergent consequences. In cortex, sustained microglial iNOS expression coincides with progressive excitatory‐inhibitory imbalance characterized by elevated glutamatergic drive, declining GABAergic tone, and neuronal activity suppression, pointing toward a maladaptive circuit restructuring. In hippocampus, microglial ARG1 and TREM2 enrichment associates with selective excitatory synaptogenesis, preserved inhibitory function, and maintained neuronal activity, indicating adaptive plasticity that may preserve cognitive capacity. The molecular mechanisms underlying these regional differences whether they reflect intrinsic microglial heterogeneity, differential cytokine milieu, or region‐specific blood–brain barrier dynamics remain to be fully elucidated and require further investigation.

## 5 Conclusions

Our findings reveal that remission from peripheral inflammation does not equate to brain immune resolution: cortical microglia in prefrontal and anterior cingulate regions sustain iNOS expression coinciding with excitatory‐inhibitory synaptic imbalance, while hippocampal microglia across DG, CA1, and CA3 subfields adopt ARG1‐dominant phagocytic phenotypes associated with adaptive synaptic remodeling. These region‐specific adaptations persist despite normalization of systemic inflammation and blood–brain barrier function, establishing microglial immune memory as a candidate substrate for neuropsychiatric comorbidities in UC.

## 6 Limitations of Our Study

Our study establishes region‐specific microglial reprogramming as a persistent feature of UC remission. We examined female mice exclusively, as sex differences in microglial biology and immune responses are well‐documented; however, male cohorts should be considered to determine whether cortical pro‐inflammatory persistence and hippocampal adaptive responses exhibit sex‐specific patterns. The DSS model recapitulates core features of human UC, including relapsing–remitting inflammation and systemic immune activation, but it does not capture the full environmental, dietary, and genetic heterogeneity that influences clinical disease trajectories. Our multimodal phenotyping approach provides a comprehensive regional characterization; yet single‐cell RNA sequencing would further resolve microglial subpopulations and identify transcriptional programs that drive cortical versus hippocampal trajectories. Our focus on microglial mechanisms was deliberately prioritized to establish the cellular and molecular basis of region‐specific neuroinflammation during remission. While we demonstrate an association between microglial phenotypes and synaptic remodeling, establishing a direct cause would require microglial depletion or selective modulation experiments. We acknowledge that CD45^int^CD11b^+^ gating can be imperfect in inflammatory contexts, as a minor subset of highly activated microglia may upregulate CD45; however, this affects only a small fraction of the total CD45^int^ population and does not substantially alter its composition. The predominant P2Y12R+ and CX3CR1+ expression profile of the population we interrogated is consistent with resident microglial identity, and CD45^hi^ infiltrating populations were analyzed separately. We propose that circulating mediators, including gut‐derived extracellular vesicles that can cross the BBB and deliver inflammatory cargo to the CNS, represent candidate mechanisms sustaining cortical microglial activation despite peripheral remission.

## Author Contributions

L.M. and I.R.D. conceived the study in collaboration with D.B., who provided the DSS‐induced colitis model. N.L. performed and monitored DSS colitis experiments. A.P.G. and L.E.V. coordinated experimental timelines. L.M. and A.P.G. designed flow cytometry panels and tissue collection protocols. L.M., A.P.G., E.W.‐E., and L.E.V. performed tissue collection, processing, and flow cytometry analysis. L.E.V. conducted confocal microscopy imaging and RT‐qPCR. L.E.V. and L.M. performed synaptosome analysis. L.M. led data interpretation, framework conceptualization, and manuscript preparation. I.R.D. supervised the project and secured funding. All authors reviewed and approved the final manuscript.

## Funding

The study was supported by institutional resources from the Otto‐von‐Guericke‐University Magdeburg and by a grant from the German Research Foundation/Deutsche Forschungsgemeinschaft (DFG, 361210922/GRK2408) to D.B.

## Ethics Statement

All animal experiments were conducted in accordance with the European Union Directive 2010/63/EU and German national regulations for the care and use of laboratory animals. Experimental protocols were approved by the local authorities (Landesamt für Verbraucherschutz, Saxony‐Anhalt, Germany) under license ID AZ 42502‐2‐1521 Uni MD. All animals were housed under pathogen‐free conditions at the central animal facility of the Medical Faculty, Otto‐von‐Guericke‐University Magdeburg, with ad libitum access to food and water. All efforts were made to minimize animal suffering and reduce the number of animals used.

## Consent

The authors have nothing to report.

## Conflicts of Interest

The authors declare no conflicts of interest.

## Supporting information


**Figure S1:** (a) Body weight fluctuations over time in control (black line), chronic colitis (red line), and chronic remission (yellow line). The period of DSS administrations and dose are indicated by blue shadowed areas. Circles in black, red and yellow represent the time points for control, chronic colitis and chronic remission groups, respectively. (b–d) Bar graphs showing levels of circulating cytokines from control mice and mice with chronic colitis or remission state. Data are shown as mean ± SEM with individual data points (*n* = 6 per group). *p* values are from one‐way ANOVA with Tukey post hoc (for parametric data) or Kruskal–Wallis with Dunn's test (for nonparametric data), as determined by Shapiro–Wilk normality testing. Exact significant *p* values are displayed above each comparison.

**Figure S2:** Representative flow cytometry gating strategy of peripheral immune myeloid (a) and lymphoid (b) cell populations of mice subjected to DSS‐induced colitis. Flow cytometry dot plots representing FSC versus SSC gate to assess size and granularity, followed by selection of single events via FSC‐A and FSC‐H. Next, a viability marker was used to positively select live cells: for myeloid panel, myeloid population was selected (CD45^hi^/CD11b^+^), followed by neutrophils (Ly6G^+^/CD11b^+^), monocyte subsets (CD11b^+^/Ly6C^High^‐Ly6C^Int^‐Ly6C^Low^), dendritic cells (DCs) (MHC‐II^+^/CD11c^+^) and classical type 2 DC population (cDC2) (CD11b^+^/CD8a^+^); for lymphoid panel, lymphocytes population (CD45^hi^/CD11b^−^), NK population (NK1.1^+^/CD3^−^), NKT cell population (NK1.1^+^/CD3^+^), T cell population (NK1.1^−^/CD3^+^), classical NK population (NK1.1^+^/NKp46^−^), type 1 innate lymphoid cells (ILC1) population (NK1.1^+^/NKp46^+^), T regs population (CD3^+^/CD25^+^), T cytotoxic population (CD4^−^/CD8a^+^), T helpers population (CD4^+^/CD8a^−^), type 2 T helpers (Th2) population (CCR6^+^/CCR3^+^), type 9 T helpers (Th9) population (FSC‐H/CCR6^+^).

**Figure S3:** Bar graphs display absolute median fluorescence intensity (MFI) values for nine microglial surface markers across groups in cortex (a–i) and hippocampus (j–r). All data shown as mean ± SEM with individual data points (*n* = 8 mice per group per experiment, repeated twice). Statistical analysis: one‐way ANOVA with Tukey's multiple comparisons (parametric data, determined by Shapiro–Wilk normality test) or Kruskal–Wallis with Dunn's post hoc (nonparametric data). Exact *p* values displayed; values > 0.9999 indicate no significant difference. Statistical significance from these analyses is summarized here to complement the z‐score heatmaps shown in Figure 2.

**Figure S4:** (a–d) Cortex and hippocampus: Fold change in Tjp1 (ZO‐1) and fos (c‐Fos) mRNA expression, normalized to *Hprt* and expressed relative to control. (e) Motor cortex (M1): Mean fluorescence intensity of iNOS and ARG1 in Iba1^+^ microglia. Data included for regional comparison with PFC and ACC (Figure 4b,c), demonstrating that iNOS upregulation during remission extends across multiple cortical subregions. Data presented as violin plots (gene expression) or bar graphs (MFI) with mean ± SEM and individual points. Statistical analysis: one‐way ANOVA with Tukey's or Kruskal–Wallis with Dunn's. *p ≤ 0.05; **p ≤ 0.01; ***p ≤ 0.001; ****p ≤ 0.0001. (f) Additional synaptosome gating strategy for inhibitory postsynaptic scaffolds: Flow cytometry plots illustrating sequential gating: synaptosomes were first identified by size (300–1000 nm) and FM4‐64FX+ membrane staining (left panel), then selected for co‐expression of pan‐synaptic vesicle markers synaptophysin and synaptobrevin‐2 (Syp/SB2+). Syp/SB2+ synaptosomes were discriminated into excitatory versus inhibitory populations based on postsynaptic receptors GluR1 (AMPA receptor subunit 1) versus NLG2 (neuroligin‐2), glycin receptor (GlyR) and gephyrin (NLG2/GlyR/Gephyrin+).

**Figure S5:** (a, b) Representative confocal immunofluorescence images of NeuN+ cells (gray) and c‐Fos+ neurons (yellow) in (a) cortical and (b) hippocampal regions across experimental groups. Individual panels of merge images from panoramic views showing c‐Fos+neuron density (Scale bar 10× image = 50 μm).

**Table S1:** Peripheral immune lymphoid cell markers.

**Table S2:** Peripheral immune myeloid cell markers.

**Table S3:** Microglia activation markers.

**Table S4:** Antibodies for Immunofluorescence analysis.

**Table S5:** TaqMan probes used for gene expression analysis.

## Data Availability

All data available upon request.
